# A Facile pH Controlled Citrate-Based Reduction Method for Gold Nanoparticle Synthesis at Room Temperature

**DOI:** 10.1186/s11671-016-1576-5

**Published:** 2016-08-15

**Authors:** Himanshu Tyagi, Ajay Kushwaha, Anshuman Kumar, Mohammed Aslam

**Affiliations:** Department of Physics, Indian Institute of Technology Bombay, Powai, Mumbai 400076 India

## Abstract

The synthesis of gold nanoparticles using citrate reduction process has been revisited. A simplified room temperature approach to standard Turkevich synthesis is employed to obtain fairly monodisperse gold nanoparticles. The role of initial pH alongside the concentration ratio of reactants is explored for the size control of Au nanoparticles. The particle size distribution has been investigated using UV-vis spectroscopy and transmission electron microscope (TEM). At optimal pH of 5, gold nanoparticles obtained are highly monodisperse and spherical in shape and have narrower size distribution (sharp surface plasmon at 520 nm). For other pH conditions, particles are non-uniform and polydisperse, showing a red-shift in plasmon peak due to aggregation and large particle size distribution. The room temperature approach results in highly stable “colloidal” suspension of gold nanoparticles. The stability test through absorption spectroscopy indicates no sign of aggregation for a month. The rate of reduction of auric ionic species by citrate ions is determined via UV absorbance studies. The size of nanoparticles under various conditions is thus predicted using a theoretical model that incorporates nucleation, growth, and aggregation processes. The faster rate of reduction yields better size distribution for optimized pH and reactant concentrations. The model involves solving population balance equation for continuously evolving particle size distribution by discretization techniques. The particle sizes estimated from the simulations (13 to 25 nm) are close to the experimental ones (10 to 32 nm) and corroborate the similarity of reaction processes at 300 and 373 K (classical Turkevich reaction). Thus, substitution of experimentally measured rate of disappearance of auric ionic species into theoretical model enables us to capture the unusual experimental observations.

**Electronic supplementary material:**

The online version of this article (doi:10.1186/s11671-016-1576-5) contains supplementary material, which is available to authorized users.

## Background

Noble metal nanoparticles have been intensively studied during the past two decades. Metal nanoparticles show optical properties of significant technological interest, including enhanced fluorescence [1, 2], non-linear optical absorbance [3], optical resonances in the near infrared region [4], and orientation-dependent plasmon excitation [5]. Among these, gold nanoparticles (AuNPs) exhibit a strong absorption of wavelengths due to surface plasmon resonance (SPR) in the visible range [6]. The first synthesis of Au colloids was reported 150 years ago by Michael Faraday using phosphorous to reduce AuCl_4_^−^ ions [7]. For efficient and effective use in applications, synthesis routes must render monodisperse AuNPs of tailored size and shape. There exist several synthesis routes [8–11], which could be used to obtain gold nanoparticles with desired structural and physical characteristics. One of the most celebrated among these has been the Turkevich method [12], wherein a mild reducing agent trisodium citrate is added to a boiling aqueous solution of HAuCl_4_ to obtain monodisperse AuNPs. Later, Frens demonstrated that the control over the size of the gold nanoparticles in Turkevich synthesis could be readily achieved by varying the relative concentration of trisodium citrate [13]. Controlling the structural characteristics of the nanoparticle needs the manipulation of kinetic and thermodynamic parameters of the systems using various additives, light and thermal energies, and their various combinations [14–16]. To achieve this control, gold nanoparticles are usually grown through a fast nucleation process followed by a diffusion-controlled growth [17, 18]. The size and polydispersity of the resulting nanoparticles are thus controlled in a way similar to the well-known LaMer model [19], which is also known as “focusing of size distribution” in the field of nonaqueous solution synthesis of nanoparticles at elevated temperatures [20]. However, from the green chemistry point of view, such nonaqueous synthesis routes are not ideal and to make AuNPs biocompatible, lengthy, and laborious process of surface functionalization is generally required [21].

The AuNPs synthesized via Turkevich approach can be size-controlled in an 8- to 100-nm range, but the polydispersity increases with particle size [13]. Besides, the standard approach yields spherical particles, but it has been shown that other geometrical shapes can be obtained by applying minimum modifications to existing protocol [21–23]. Controlling the pH and precursor to reductant concentration ratio is an integral part of such modifications. Herein, we study the effect of the pH on the synthesis of gold nanoparticle at *room temperature*. The synthesis of gold nanoparticles via room temperature approach is simple and convenient and gives a narrow particle size distribution (11.7 ± 2.2 nm). There exists an optimal pH for every colloidal solution at which the particles formed are fairly stable and monodisperse without an extra stabilizing agent. At pH values other than the optimal value, there is uncontrolled nucleation and growth which results either in anisotropic shape of particles or in coagulation of particles, thus giving polydispersity in size distribution. The synthesis of AuNPs via Turkevich method involves several steps among which citrate reduction of Au^3+^ species is the first rate-determining step. We calculate the reaction rate (*k*_c_) of this step at room temperature in Turkevich approach. It is found that this rate depends critically on the pH and precursor ratio of the reaction mixture. The large value of *k*_c_ (7655 M^−1^ s^−1^) for the optimized case of 2:1 citrate to AuCl_3_ (pH 5) helps in quick synthesis of AuNPs at room temperature. We use the different rate values in simulation program developed in lines of theoretical model incorporating nucleation, growth, and aggregation processes. The simulation predicts that the mean particle size under various experimental conditions and theoretical predictions agrees well with the corresponding experimentally obtained sizes of AuNPs. Such comparison further substantiates the similarities of intermediate reaction steps in the Turkevich process at 373 K and room temperature. The room temperature synthesis of AuNPs via citrate-based reduction of gold(III) chloride represents a bio-friendly approach and gives us an insight into pH controlled reduction of Au^3+^ ions. The study reveals a novel and easy way to manipulate Turkevich like method carried out at room temperature. Though the literature regarding citrate based reduction of AuCl_3_ to obtain AuNPs is quite extensive, our method introduces a fundamental change in existing method in terms of temperature conditions.

## Methods

**Scheme 1:**
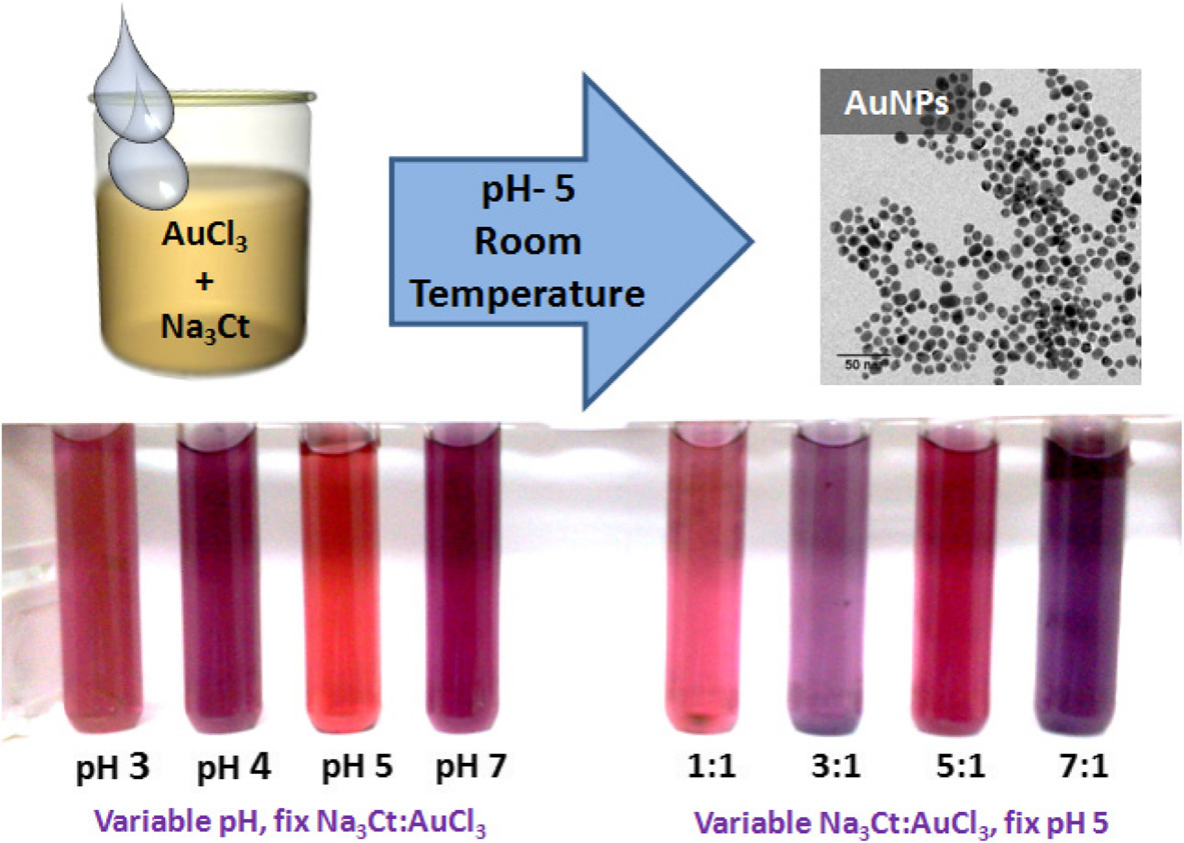
Scheme showing preparation of AuNPs at optimized pH via citrate-based reduction process carried out at room temperature. The varying color of prepared AuNP solutions shows the role of optimized pH and reactant ratios

Gold(III) chloride 99 % (ACROS Organics, NJ, USA) and trisodium citrate dihydrate (Merck Limited) were used as received without further purification. A typical synthesis involves taking a fixed concentration ratio of trisodium citrate to gold trichloride. Appropriate volumes of 10 mM solution of trisodium citrate and 0.5 mM solution of gold trichloride were mixed to get a desired molar concentration ratio such that the total volume of the solution is 10 ml. The solution of the sodium citrate and gold chloride is stirred at room temperature for up to 48 h under ambient conditions in a beaker covered with glass plate. The solution color shows a transient color change during the stirring and becomes stable in 2–3 h. The dilute HCl or NaOH is added to control the solution pH. Such studies were carried out for precursor ratios of 1:1, 2:1, 3:1, 5:1, 7:1, and 9:1 with different pH values ranging from 3 to 9 (Scheme 1). In pH range other than 2.5 < pH < 9, the solution remains colorless for days indicating no gold nanoparticles formation at such low/high pH conditions.

Optical absorption spectra were taken using Lambda 950 (Perkin Elmer) in the wavelength range 200–800 nm at room temperature using quartz cell. Transmission electron microscopy (TEM) was carried out using JEOL JEM-2000F. Samples for TEM characterization were prepared by placing a drop of gold colloidal solution on carbon coated copper grid and dried under IR lamp. For further characterization, Fourier transform infrared spectroscopy (FTIR) was performed using MAGNA 550, Nicolet Instruments Corporation, USA. X-ray diffraction (XRD) of gold nanoparticles was carried out using “PANalytical Xpert PRO.” XRD sample was prepared by drop-casting colloidal gold nanoparticles on glass slides.

## Results and Discussion

### Reaction Time of AuNP Synthesis

A typical Turkevich synthesis of AuNPs at 373 K takes 20 min for the characteristic red wine color to appear in the solution. There have been recent reports where researchers have been able to decrease the reaction temperature up to 343 K by optimizing the pH and reactant ratio of reaction mixture [24]. According to Turkevich et al. [12], the solution temperature (373 K) plays an important role for the gold nanoparticle formation, and for every decrease of 10 K there is a twofold increase in time period necessary for the completion of reaction. This implies that at 300 K, reaction shall take around 2600 min to complete. However, if appearance of red wine color is to be taken as an indicator of near completion of reaction, for a particular case of molar ratios 2:1 and 5:1 (pH 5), the reaction is over within 8 h. In contrast to this, for other molar ratios, no further change in blue-violet color is observed after 24 to 48 h. Interestingly, these observations suggest that at room temperature, a fivefold reduction in reaction time is possible through adjustments of precursor ratio and initial pH condition, i.e., at pH 5, 2:1 and 5:1 precursor molar ratio.

### Synthesis and “Colloidal” Stability of Gold Nanoparticles

**Fig. 1:**
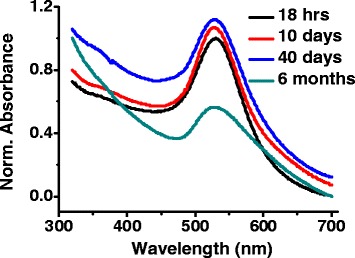
Normalized UV-vis spectra of gold nanoparticles synthesized via room temperature approach (RT) showing long-term stability of colloidal suspension (concentration ratio of citrate and gold(III) chloride is 5:1 at pH = 5)

After the synthesis of gold nanoparticles via room temperature approach, the UV-vis spectroscopy was performed at different time intervals (days to months) to check the stability of the colloidal suspensions. Figure 1 shows the corresponding UV-Vis data of gold nanoparticles taken after 2 h, 18 h, 10 days, 40 days, and 6 months, respectively. The particles synthesized via room temperature approach initially show a broad peak which indicates aggregates of various sizes at the initial growth stages. It might be possible that, at this stage, the nuclei particles have multiple sizes, in a manner similar to that suggested by the nanowire growth model [25]. The reaction completes within 18 h and UV-vis absorption spectra for the room temperature synthesized samples after 10 and 40 days of aging illustrate a narrow and sharp absorption peak which is a clear indication of the existence of the stable nanoparticles in colloidal form. The SPR peak of these AuNPs synthesized via room temperature approach broadens up in a 600- to 700-nm wavelength range after aging of 6 months which indicates presence of aggregates in AuNP sol. Hence, the particles synthesized via room temperature approach remain stable for more than a month in colloid form.

### Nucleation and Growth of AuNPs (Case of 300 K)

**Fig. 2:**
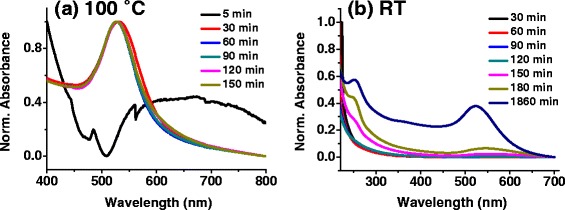
Time dependent normalized UV-vis spectra of gold nanoparticles. **a** Classical Turkevich approach (100 °C) at pH = 5. **b** Room temperature approach (RT) at pH = 5

According to Turkevich et al., the solution temperature plays an important role in the synthesis of gold nanoparticles [12]. When the temperature is lowered by 10 °C, the necessary time for the completion of reaction increases by a factor of 2; however, the mean particle size and the root mean square deviation narrows down with a decrease in synthesis temperature. In our approach, the reaction rate is slow due to ambient conditions (room temperature) though the typical “ruby-red” color appears within 3 h depending upon the pH control. The time dependent UV-vis absorption study is performed for different precursor ratios like 2:1, 3:1, 5:1, and 7:1 at their optimal pH condition to monitor the nanoparticle nucleation and growth. Figure 2a, b illustrates such plots for the precursor ratio of 5:1 (pH 5), synthesized via conventional Turkevich approach (Fig. 2a) and room temperature approach (Fig. 2b), respectively. As opposed to quick synthesis of AuNPs at 100 °C (within 30 min), the flat curves (no absorbance peak, Fig. 2b) for synthesis at room temperature suggest that the growth is not initiated till 120 min; however, the nucleation might have happened (pale yellow color changes to colorless). After 2 h, the appearance of some particles is seen (pink color of the solution), and the broad absorption peak suggests a broader size distribution during the initial growth stage [21]. As the time increases, the FWHM of the UV-absorption peak decreases and there is no more change in peak shape after 30 h. This suggests that the particles are uniform in size and the growth is complete. Hence, at room temperature the reaction rate is slower, and therefore, reduction of gold salt goes slower in the presence of citrate. It might be possible that the role of citrate as a reducing agent takes a back seat at room temperature. Meanwhile, the citrate stabilizes the gold which restricts the nanoparticle growth to a critical size, and hence, the nanoparticles are more stable. The existence of optimal pH is a consequence of increase in the reactivity of gold complexes and interparticle attraction with decreasing pH [10, 21, 26].

### Role of pH for the Control Over Nucleation and Growth of Nanoparticles

**Fig. 3:**
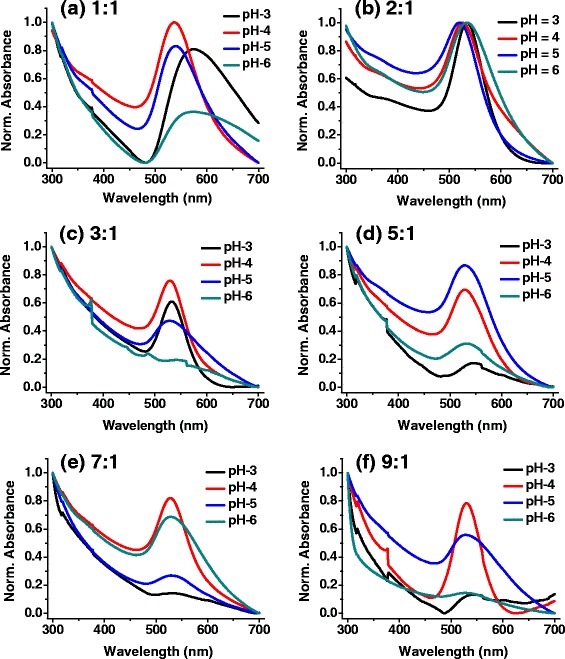
Normalized UV-vis spectra of gold nanoparticles with different sodium citrate and AuCl_3_ at different pH values. **a** 1:1 ratio, **b** 2:1 ratio, **c** 3:1 ratio, **d** 5:1 **e** 7:1, **f** 9:1. Normalization has been done in extended range to show contrast in quality of samples

**Fig. 4:**
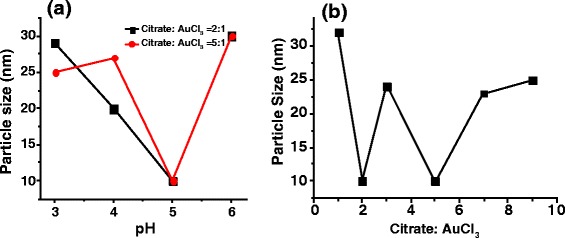
**a** Particle size with their respective pH is plotted at fixed (2:1 and 5:1) concentration ratios. **b** Variation in mean particle size with citrate to AuCl_3_ ratio. Citrate to AuCl_3_ ratio should be 2:1 or 5:1 to obtain small particle size

Solution pH plays an important role for the size and shape control of the gold nanoparticles [10, 21, 24]. Ji et al. studied the growth kinetics of nanocrystalline gold at 373 K in citrate reduction [21]. They found that the shape and size dependence is strongly influenced by the pH of the reacting mixture. A two-step mechanism of rapid nucleation and slow growth by monomer diffusion (pH >6.5) and a three-step mechanism with interparticle ripening as the last step (pH <6.5) were proposed, thus implying a strong pH dependence. We synthesized gold nanoparticles at room temperature taking different pH conditions for various concentration ratios of citrate to gold precursor. We have taken here six different concentration ratios 1:1, 2:1, 3:1, 5:1, 7:1, and 9:1 for citrate/gold and controlled the pH value of the solution from 2 to 12. The UV-vis spectra of these samples (Fig. 3) show that each mole ratio has its own optimal pH for a better particle size distribution. The 1:1 surfactant/salt ratio for a control of pH 3 and 6 reveals very broad UV absorbance curve (Fig. 3a). Interestingly, pH 4 controlled solution of same mole ratio show a sharp absorbance peak approximately at 540 nm. Comparatively, the pH 4 for the above concentration has the most intense and sharp peak. Therefore, concentration ratio 1:1 has an optimal pH 4 which renders a narrower size distribution. When citrate to gold ratio is 5:1, the SPR is centered at 530, 532, 521, and 536 nm for pH values of 3, 4, 5, and 6, respectively (Fig. 3c). It means that for pH values lower and higher than the optimal pH (pH 5) condition, the size of the nanoparticles is bigger and the particle size distribution is non-uniform. Hence, the initial pH value of the solution is one of the critical size deciding parameters of the gold nanoparticles. The relation between sizes of the gold nanoparticle with respect to the pH of the solution is represented as Fig. 4a. The particle size and polydispersity is lowest (11.7 ± 2.2 nm) for the optimal pH 5, wherein the narrow SPR band at minimum wavelength is observed.

In order to explain the effect of pH on reaction in Turkevich process, we must look at role of H^+^ and OH^−^ ions in the standard reaction mechanism [12, 27]. In this reaction, there are three parameters which are responsible for the controlled synthesis of gold nanoparticles, namely, concentration of gold chloride and sodium citrate, and the pH of the solution. The reaction takes place in various steps in between the final product and reactants. First, gold chloride decomposes into gold and chloride ions, while sodium and citrate ions are produced by the dissolution of sodium citrate. During the reaction, trisodium citrate plays the role of reducing as well as stabilizing agent and if concentration relative to gold precursor is high, it act as a buffering agent also [21, 22].

The citrate gets oxidized and produces dicarboxy acetone; however, before occurrence of the above reaction, the pH value of the solution has been controlled using HCl/NaOH. In the first case, when dilute HCl (HCl = H^+^ + Cl^−^) is added to lower the pH of the solution, HCl provides the H^+^ ions to the solution. Simultaneously, the gold(III) chloride reduced into gold(I) chloride (Eq. 2) by accepting the electrons from the citrate oxidation reaction (Eq. 1). Various research groups have suggested the formation of an intermediate pentacoordinate complex of Au^3+^ species with dicarboxyacetone (DCA) which subsequently decarboxylates to give Au^+^ species during the reaction [28]. The rate of such a ligand exchange reaction which yields pentacoordinated complex is temperature dependent and is expected to be slow at room temperature when compared to 373 K.

1 $$ {\left({}^{-}{\mathrm{OCOCH}}_2\right)}_2\mathrm{C}\kern0.5em \left(\mathrm{O}\mathrm{H}\right){\mathrm{CO}\mathrm{O}}^{-}\to {\left({}^{-}{\mathrm{OCOCH}}_2\right)}_2\mathrm{C}=\mathrm{O}+{\mathrm{CO}}_2+{\mathrm{H}}^{+}+2{\mathrm{e}}^{-} $$

2 $$ {\mathrm{AuCl}}_3+2\kern0.5em {\mathrm{e}}^{-}\to \mathrm{AuCl}+2{\mathrm{Cl}}^{-} $$

AuCl so formed gets converted into gold nanoparticles and gold(III) chloride as:

3 $$ 3\mathrm{AuCl}\to 2\mathrm{A}\mathrm{u}+{\mathrm{AuCl}}_3. $$

As the H^+^ ions increase in the solution after addition of HCl, the availability of electron to reduce the gold(III) chloride becomes lesser and reaction rate becomes slower in this case. In accordance with Le Chatelier’s principle [29, 30] and pK_a1_ being 3.1 for citrate [31], the tendency of citrate to oxidize decreases at a low pH (<3). Hence, there is a lack of electron for the reduction of gold(III) chloride at such minimum pH. For this pH condition, no color change is observed in the solution, and hence, we conclude that reaction is not taking place at very low pH conditions.

Similarly, for higher pH values (pH >9), the diluted NaOH (NaOH = Na^+^ + OH^−^) has been added to provide the OH^−^ ionic species in the solution. Hydroxyl ions directly react with AuCl_4_^−^ and convert it into AuCl_3_(OH^−^), hence making it less reactive. At higher pH (>9) values, there is no color change in the solution, and hence, the reaction does not take place at higher pH conditions [10, 27]. It is important to note that while best particle size distribution was obtained at pH >6 by Ji et al. [21], pH should be lesser than 6 to get monodisperse AuNPs if citrate-based reduction is performed at room temperature. At high temperature, both nucleation and growth are faster processes in Turkevich reaction and a high pH restricts the nucleation and growth processes rendering monodisperse particles. In comparison, when reaction is carried out at room temperature, nucleation in our studies is a slow process at higher pH conditions, and simultaneous nucleation and growth may introduce inhomogeneity in AuNP sizes. However, in our case interestingly at low pH conditions, fraction of AuCl_4_^−^ in reaction mixture increases which improves the nucleation rate and results in formation of monodisperse particles. The hydroxylation of Au^3+^ species thus determines the size distribution of AuNPs at room temperature in a quite contrary way as compared to high temperature reactions. Moreover, the tendency of citrate to oxidize decreases with decrease in pH, and hence, its role as a stabilizing agent becomes more prominent. A better nucleation rate achieved at room temperature via lowering of pH and slow growth thus yields monodisperse AuNPs.

**Fig. 5:**
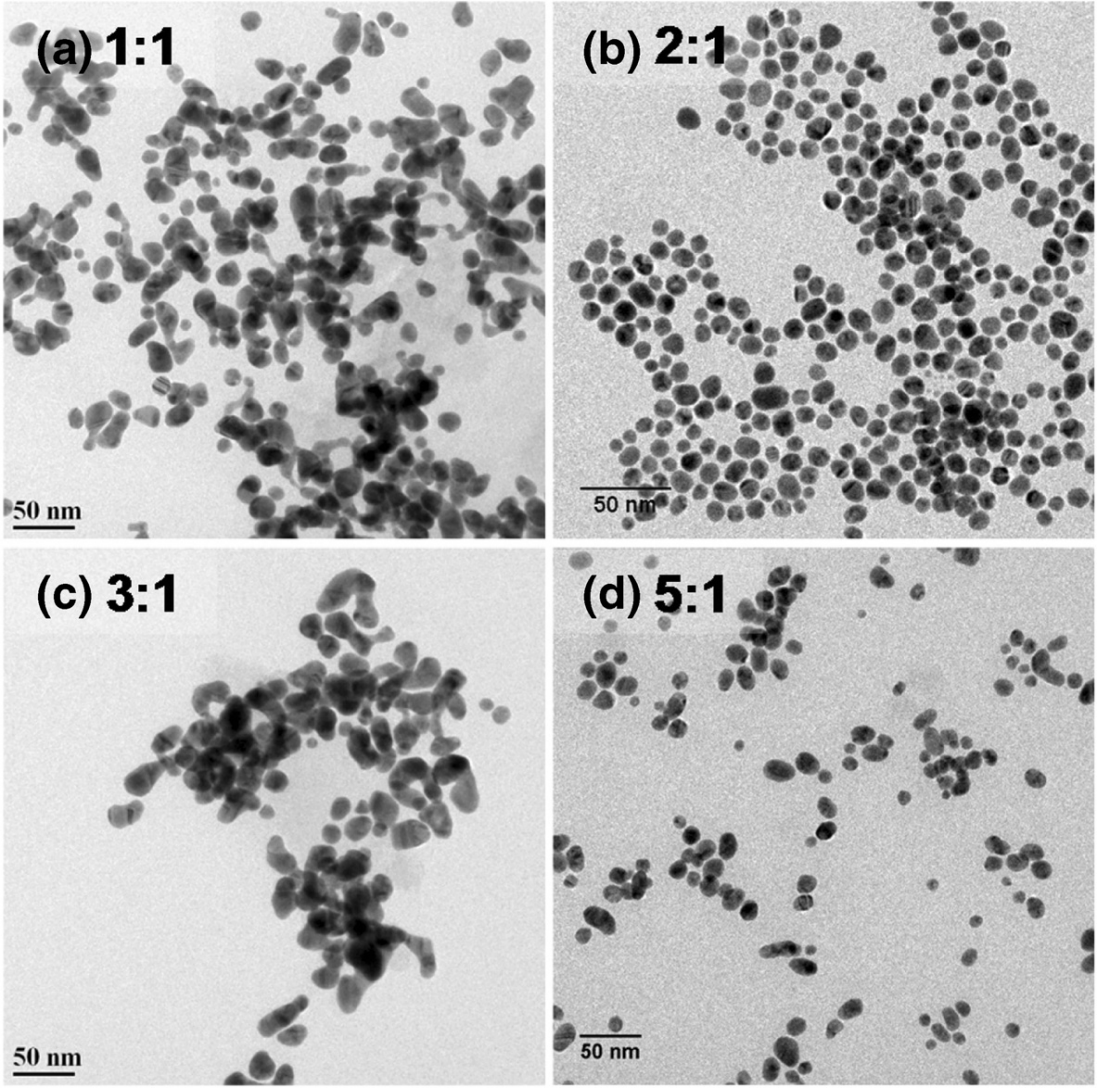
TEM images of AuNPs formed with various citrate to AuCl_3_ ratio (**a**) 1:1, (**b**) 2:1, (**c**) 3:1, and (**d**) 5:1. The pH value was fixed at 5 for all cases. Agglomeration of particles takes place for molar ratios other than 2:1 and 5:1 (for TEM images with particle distribution on a larger scale, see Additional file 1: Figure S1)

The concentration ratio Na_3_Ct:AuCl_3_ is another parameter which controls the average particle size and the dispersion significantly. The optimal pH (at which particles have narrow size distribution) for various surfactant/salt concentration ratio is found to be either 4 or 5. Thus, irrespective of the concentration ratio we take, pH should be adjusted in the range 4–5 to obtain monodisperse gold nanoparticles. The effect of the concentration ratio on the particle size is evident from TEM images of various samples (Fig. 5). The particle size observed in experiments lies in the size range 10 to 30 nm (Fig. 4b). Thus, concentration ratio Na_3_Ct:AuCl_3_ significantly affects the final size distribution. In citrate based reduction of AuCl_3_, typically an initial phase of nucleation with simultaneous generation of DCA is followed by growth of nuclei which is initially slow but faster in the final phase of reaction [32]. It is known that adsorption of Au^+^-DCA complex assists the growth of Au nuclei during nucleation and growth process [12, 33, 34]. There are two possible growth mechanisms proposed by researchers for Turkevich synthesis viz. nanowire intermediate-based growth model and a model based on initial phase of nucleation followed by slow and fast phases of growth [21, 32, 33, 35, 36]. Even though nanowire growth model has been investigated thoroughly by researchers, it is suspected that nanowire structure is an artifact arising from drying and interaction of electron beam with sample during TEM characterization [33]. However, irrespective of the model, we choose to describe the formation of AuNPs, it is known from experiments that citrate to AuCl_3_ ratio in range 2 to 5 yields smaller sized particles and nucleation is faster at those ratios [26]. The sweet spot of appropriate citrate to AuCl_3_ ratio is related to pH-dependent speciation of [AuCl_4_]^−^ and citrate ions (Eqs. 4 and 5) [37].

4 $$ {\left[{\mathrm{AuCl}}_4\right]}^{-}+x{\mathrm{H}}_2\mathrm{O}\leftrightarrow {\left[{\mathrm{AuCl}}_{4-x}{\left(\mathrm{O}\mathrm{H}\right)}_x\right]}^{-}+x{\mathrm{H}}^{+}+x{\mathrm{Cl}}^{-} $$

5 $$ {\mathrm{Cit}}^{3-}+x{\mathrm{H}}^{+}\leftrightarrow {\mathrm{H}}_x{\mathrm{Cit}}^{\left(3-x\right)-} $$

The speciation of [AuCl_4_]^−^ and citrate ions in combination with formation of dicarboxyacetone is thus likely to decide nucleation in initial stages of reaction. Even though excess of citrate ions should increase the nucleation in initial stages, coupling of Eqs. 4 and 5 shows that excess of citrate ions also decreases number of more reactive [AuCl_4_]^−^ species. Hence, pH as well as concentration of citrate ions must be optimized for faster nucleation and to obtain monodisperse and small AuNPs. For citrate to AuCl_3_ ratios greater than 5, nucleation is slow, while for ratio values <2, insufficient concentration of citrate is not able to completely reduce the precursor (and cap the particles) resulting in agglomeration of particles (Fig. 5) [26]. The formation of monodisperse AuNPs obtained by us for citrate to AuCl_3_ ratio 2:1 and 5:1 in comparison to other ratios is thus in accordance with literature. Since fast nucleation is critical to the formation of monodisperse AuNPs in our method (where citrate reduces the AuCl_3_ at room temperature), adjusting citrate to AuCl_3_ ratio below 5 is crucial to obtain monodisperse and small AuNPs. However, for citrate to AuCl_3_ ratio of 3:1, we obtain polydisperse and larger AuNPs compared to the case of 5:1 and 2:1 ratios. It is possible that for the case of 3:1 ratio, the citrate concentration neither is large enough to produce Au(0) from Au(III) species nor is it less enough to have more reactive [AuCl_4_]^−^ species in reaction mixture.

**Fig. 6:**
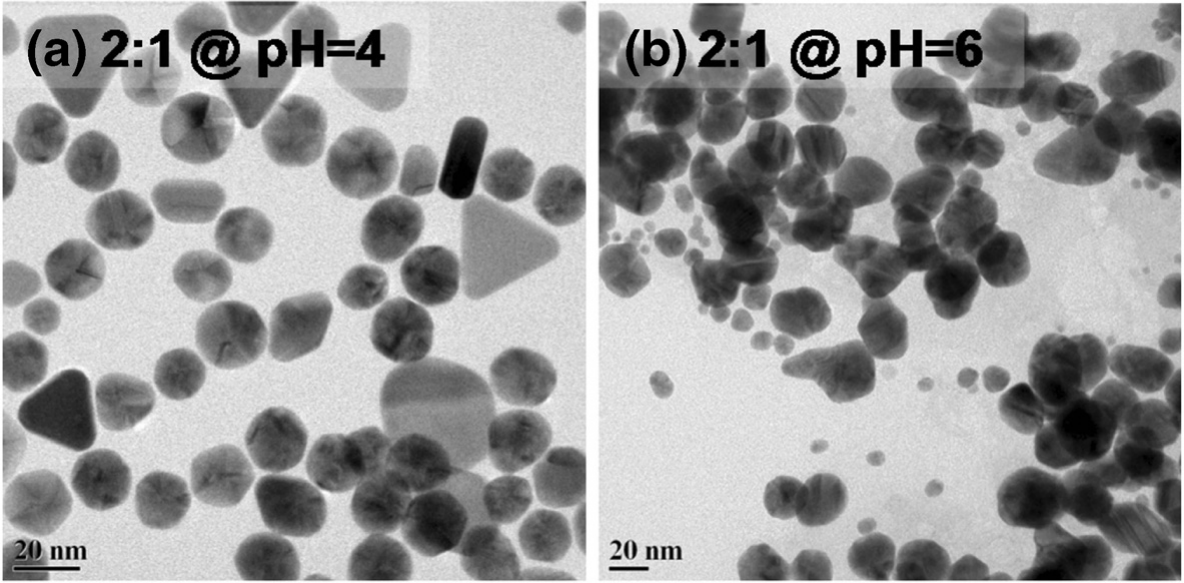
Citrate to AuCl_3_ ratio 2:1 case **a** formation of large sized, anisotropic, and rlydisperse particles at pH = 4. **b** Agglomeration of particles in pH = 6 condition

**Fig. 7:**
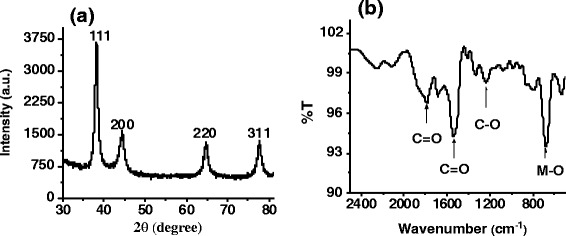
**a** X-ray diffraction pattern of drop-casted film of gold nanoparticles. **b** FTIR spectrum of the gold nanoparticles

The TEM images of particles at the different pH values 4 and 6 (fixed 2:1 precursor ratio) show anisotropy in particle shape (Fig. 6a) as well as conjoining of AuNPs (Fig. 6b) due to thermodynamically unstable reaction conditions. However, at optimal pH (Fig. 5b), the particles are monodisperse (11.7 ± 2.2 nm). To verify the presence of gold nanoparticles in colloidal solution, the FTIR spectrum was taken (Fig. 7b). The peak at 677 cm^−1^ is assigned to the M-O (M = metal; O = oxygen) stretching vibration, which confirms the capping of gold nanoparticles by citrate ions via RCOO^−^ → Au coordination [38]. The peak at 1790 cm^−1^ indicates the presence of ketonic carbon-oxygen double bonds which confirms the formation of dicarboxy acetone in reaction. The presence of citrate in AuNP solution is signified by the peaks corresponding to R-CO_2_ stretching and C-O stretching at 1540 and 1236 cm^−1^, respectively. X-ray diffraction from a drop-casted thick film of colloidal gold nanoparticles on glass substrate show the peaks at 38.7°, 45.5°, 67.1°, and 78.1° corresponding to the 111, 200, 220, and 311 planes (Fig. 7a) in gold nanoparticles.

### Mechanism of Nucleation and Growth (at Room Temperature)

The simulation models based on DLVO theory and Monte Carlo approach where coulombic charges play the most crucial role, are not suitable to model Turkevich synthesis since this is not a burst nucleation process (followed by Ostwald ripening) [32, 35]. It rather involves simultaneous nucleation, growth, and aggregation processes whereby the particle surfaces catalyze the growth, and the number of particles affects the course of chemical reactions. The theoretical model proposed by Sanjeev et al. successfully takes into account these simultaneous processes and we used it to understand the nucleation and growth process of gold nanoparticles at room temperature [39, 40]. A simulation code based on the algorithms necessary to model theoretical predictions has been written for this purpose.

While the Turkevich synthesis has been successfully modeled for reactions at 373 K temperature [41], there are no reports on prediction of particle size under room temperature conditions. We assume the similarity of reaction steps at 373 and 300 K. The important rate limiting steps for the room temperature synthesis process which could be modeled as per literature reports [12, 41] with the appropriate modification are:

6 $$ T+C=M+S\ \left(\mathrm{rate}\kern0.5em \mathrm{constant}\kern0.5em ``{k}_{\mathrm{c}}"\right), $$

7 $$ 3M=T+\mathrm{particle}\left(\mathrm{rate}\kern0.5em \mathrm{constant}\kern0.5em ``{k}_{\mathrm{h}}"\right), $$

8 $$ 3M=\mathrm{nucleus}+T\left(\mathrm{rate}\kern0.5em \mathrm{constant}\kern0.5em ``{k}_{\mathrm{n}}"\right), $$

9 $$ S=D\left(\mathrm{rate}\kern0.5em \mathrm{constant}\kern0.5em ``{k}_{\mathrm{s}}"\right), $$

10 $$ D+4T=4M+\mathrm{products}\left(\mathrm{rate}\kern0.5em \mathrm{constant}\kern0.5em ``{k}_{\mathrm{d}}"\right), $$

where, *T* = Au^3+^ species, *C* = citrate ions, *M* = Au^+^ species, *S* = dicarboxy acetone, and *D* = acetone.

The kinetics of reaction at room temperature will be different when compared to classical Turkevich synthesis. This implies that the values of rate constants (of intermediate reactions) at 373 and 300 K are different. Moreover, we account for reduction of AuCl_4−x_(OH)_x_ species by assuming experimentally obtained rate constant “*k*_c_” to be average of all Au^3+^ reductions by citrate ions. A similar assumption is made for AuCl_2−x_(OH)_x_ species while incorporating “*k*_h_” and “*k*_n_” in our calculations.

We used the following parameteric values to simulate different experimental conditions:

Temperature = 300 K; *k*_n_ = (1.67 × 10^9^)*N*_avg_ M^−5^ L ^−1^ s ^−1^; *k*_h_ = 1.8 × 10^−3^ cm^−2^ L^−1^ s^−1^; *k*_s_ = 1/1300 s^−1^; *k*_d_ = 400 M^−1^ s^−1^; *k*_c_ = variable (in M^−1^ s^−1^); *T* = conc. of AuCl_3_ (variable); and *C* = conc. of trisodium citrate (variable).

**Fig. 8:**
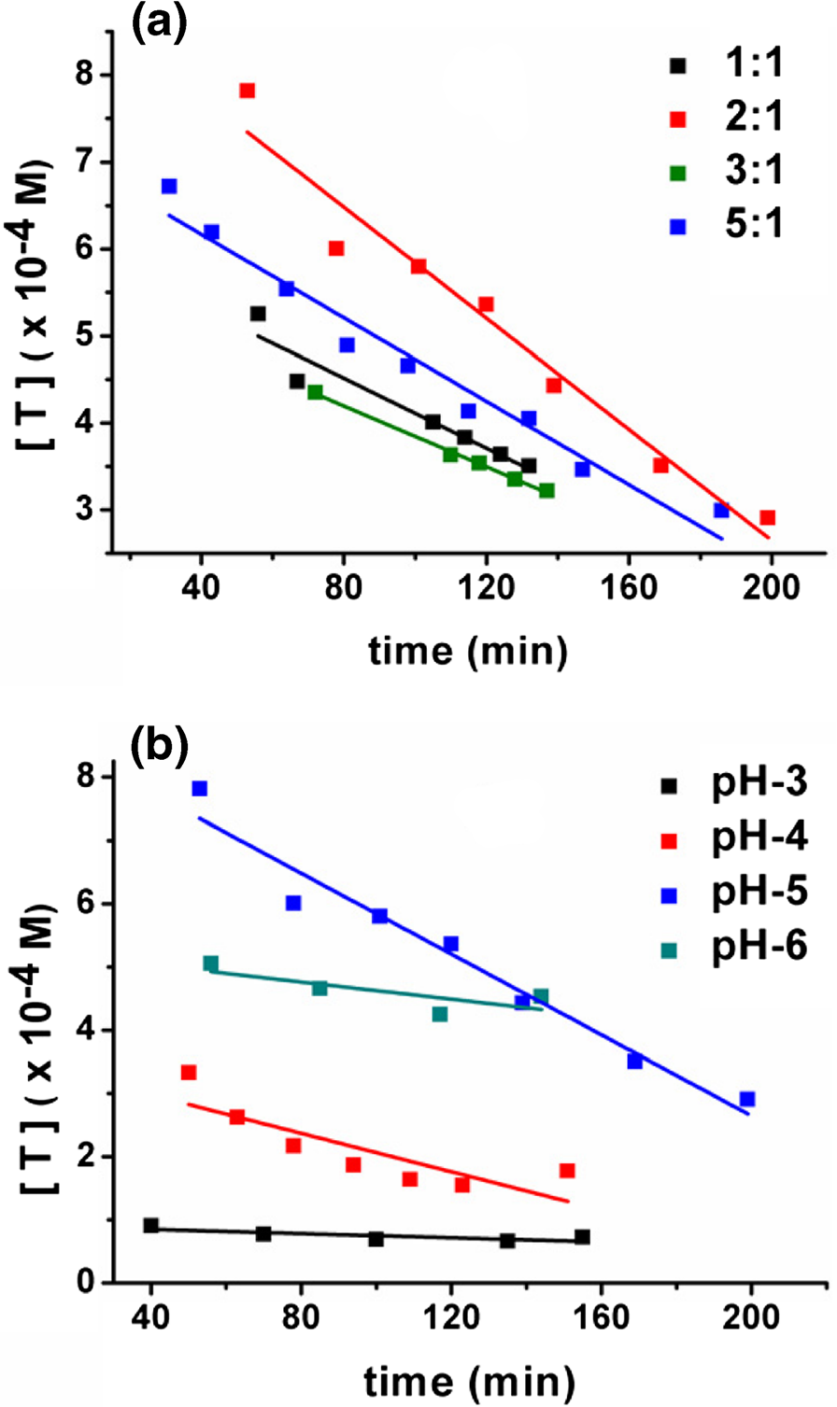
Time dependent concentration of Au^3+^ species with linear fits for **a** samples with various citrate to AuCl_3_ ratio. **b** Samples with various pH having constant citrate to AuCl_3_ ratio 2:1

The new rate constants are temperature dependent and vary accordingly for different processes [35, 42, 43]. The rate constant “*k*_c_” is related to the very first reaction step in whole process and hence any change would significantly affect subsequent reaction steps. As our experimental results exhibit a strong dependence on pH variation, we investigated the pH dependence of “*k*_c_”. In initial stages, due to inherent slowness of the reaction, concentration of Au^3+^ species (*T*) is expected to vary as:

11 $$ \frac{\mathrm{dT}}{\mathrm{dt}} \approx -{k}_{\mathrm{c}}\left[T\right]\left[C\right] $$

and we thus obtain “*k*_c_” by calculating the concentration of Au^3+^ species at different intervals during the reaction. The characteristic UV absorbance peak in the wavelength range 200–230 nm is a signature of Au^3+^ species [10, 27]. The absorbance of Au^3+^ species (and thus the [*T*]) is found to vary in a linear fashion with time and a linear fit (Fig. 8 and Eq. 12) is used to determine “dT/dt.” The saturation of Au^3+^ absorbance peak below time “*t*” = 40 min is observed; since the reaction is slow, we can use absorbances at *t* >40 min to estimate “*k*_c_”. For a given time coordinate “*t* = *t*_1_,” absorbance is then used to determine “[*T*(*t*_1_)]” and corresponding “[*C*(*t*_1_)]”. This calculation of “*T*” and “*C*” holds true under the assumption that the reduction of Au^3+^ by citrate is dominant process in initial stages of reaction. The equations used in “*T*” and “*C*” calculation are:

12 $$ \left[T\right]=\mathrm{absorbance}/\left(\varepsilon l\right), $$

13 $$ \left[C\left({t}_1\right)\right]={\left[C\right]}_{\mathrm{initial}}\hbox{--} 1.5\left({\left[T\right]}_{\mathrm{initial}}\hbox{--} \left[T\left({t}_1\right)\right]\right), $$

14 $$ {k}_{\mathrm{c}}\approx -\frac{\mathrm{dT}}{\mathrm{dt}}/\left(\left[T\left({t}_1\right)\right]\left[C\left({t}_1\right)\right]\right), $$

where, *ε* = 11900 cm^−1^ M^−1^ (absorptivity of Au^3+^ ions determined from the absorbance of a known concentration of AuCl_3_), *l* = 1 cm (path length), and [*C*]_initial_ and [*T*]_initial_ are initial concentrations of citrate and Au^3+^ ions, respectively, in a given experiment. The “*k*_c_” values for various reaction conditions are given in Tables 1 and 2.

**Table 1:** Data comparing simulated and experimental mean particle size for variable ratio (fixed pH = 5) case. The experimental trend in size variation is in close conformity with mean particle size from simulation. The precursor ratio 2:1 and 5:1 give minimum sized particles

Citrate to AuCl_3_ ratio	1:1	2:1	3:1	5:1
*k*_c_ (M^−1^ s^−1^)	3563	7655	1444	1721
Plasmon peak (nm)	539	521	529	521
Experimental particle size (nm)	32	10	24	10
Simulated particle size (nm)	25	12	21	13

**Table 2:** Data comparing simulated and experimental mean particle size for variable pH (fixed ratio 2:1) case. The experimental trend in size variation is in close conformity with mean particle size from simulation

pH	3	4	5	6
*k*_c_ (M^−1^ s^−1^)	3750	9605	7655	716
Plasmon peak (nm)	535	525	521	537
Experimental particle size (nm)	29	20	10	30
Simulated particle size (nm)	25	10	12	108

The pH dependence at a given ratio 2:1 of mean AuNP size obtained from simulation agrees well with the one observed experimentally (Table 2). The predicted values of mean AuNP diameter at pH 3 and pH 5 are in good agreement with the ones obtained from UV-vis spectroscopy and TEM data. In sharp contrast to all other pH values, the pH 6 case estimates a simulated size (108 nm) which differs significantly from experimental size (30 nm). This might be due to aggregation processes in reaction where particles are conjoined together (Fig. 6b). The simulation strategy as proposed herein, or to the best of our knowledge in the literature, does not take into account the conjoining of particles. Instead, it will account the conjoined particle as a single big size particle. Furthermore, at pH 4 condition also we find an apparent disagreement between experimental (20 nm) and predicted particle size (10 nm). The deviation can be attributed to evolution of anisotropic particles as seen in TEM image (Fig. 6a). The simulation calculates volume of particles and assuming a spherical shape yields its diameter. Hence, the apparent anomaly in diameter stems from anisotropic particles which will have a higher particle size compared to spherical geometry for same volume. When we compare results for fixed pH (pH = 5) and variable precursor ratios, the experimental and simulated results agree satisfactorily (Tables 1 and 2). The predicted particle size (tens of nm) for pH values other than 4–5 is also in agreement with experiments. It is to be noted that predicted particle sizes are well within the range of sizes obtained experimentally. The trends of increase and decrease in mean particle size with pH and precursor ratio variation match the experimental data. The agreements between experimental findings and results from simulation suggest that the theoretical model that incorporates simultaneous nucleation, growth and aggregation in a synthesis holds well for the room temperature process. Moreover, the effect of pH is accurately accounted by calculating rate constant “*k*_c_” for each case.

## Conclusions

The Turkevich synthesis of gold nanoparticles at *room temperature* is explained, and the role of the initial pH of the solution and the concentration ratio of sodium citrate to the gold chloride is discussed in detail. The study on role of initial pH of the solution of AuCl_3_ and trisodium citrate for size control shows that at optimal pH, the particle size is uniform and the particles are monodisperse. At pH values lower and higher than the optimal pH for a given precursor solution, non-uniform shape and size of the nanoparticles is obtained. The long shelf life of the gold nanoparticles without using any stabilizing or capping agent is the main outcome of this room temperature synthesis approach, and the nanoparticles remain in colloidal form for a year at room temperature. We used modified nucleation-growth model of standard Turkevich reaction to simulate our reaction and included the appropriate variations in kinetic constants using experimental results. The agreement between mean particle size from simulation and the experiments show that the reaction steps in room temperature reaction and standard Turkevich reaction are essentially same.

## Additional file

Additional file 1: TEM images depicting large scale distribution of particles, variation of LSPR wavelength with reactant ratio and pH, Zeta potential at various pH conditions and particle size distribution graphs are given in additional file. (DOCX 1453 kb)
