# Supplementary material for: A Facile pH Controlled Citrate-Based Reduction Method for Gold Nanoparticle Synthesis at Room Temperature
Source: Nanoscale Res Lett. 2016 Aug 15;11:362. doi: 10.1186/s11671-016-1576-5 (PMC4987575; doi:10.1186/s11671-016-1576-5)
Supplement: Additional file 1: — TEM images depicting large scale distribution of particles, variation of LSPR wavelength with reactant ratio and pH, Zeta potential at various pH conditions and particle size distribution graphs are given in additional file. (DOCX 1453 kb) [file 11671_2016_1576_MOESM1_ESM.docx]

**Additional file 1**

for

**A facile pH controlled citrate based reduction method for gold nanoparticle synthesis at room temperature**

Himanshu Tyagi, Ajay Kushwaha, Anshuman Kumar and Mohammed Aslam*

Department of Physics

Indian Institute of Technology Bombay, Powai, Mumbai-400076, India.

**
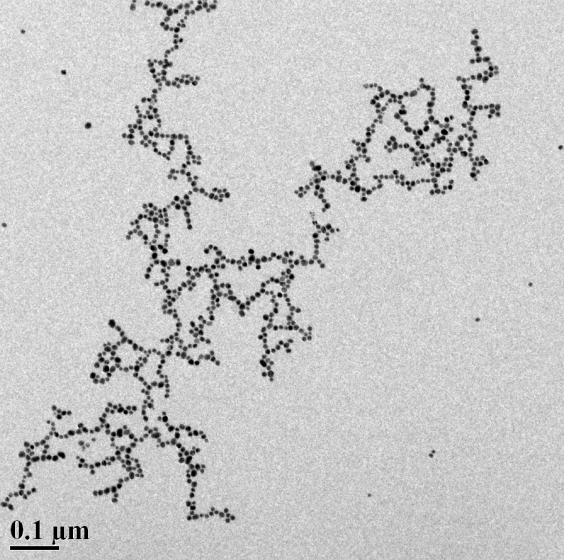

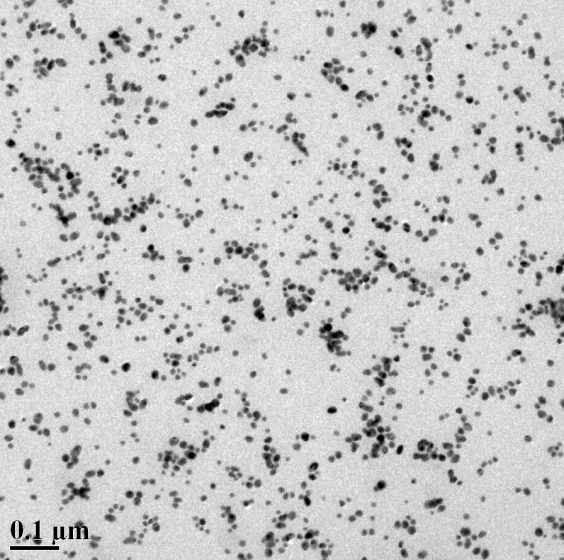
**

**(b)**

**(a)**

**
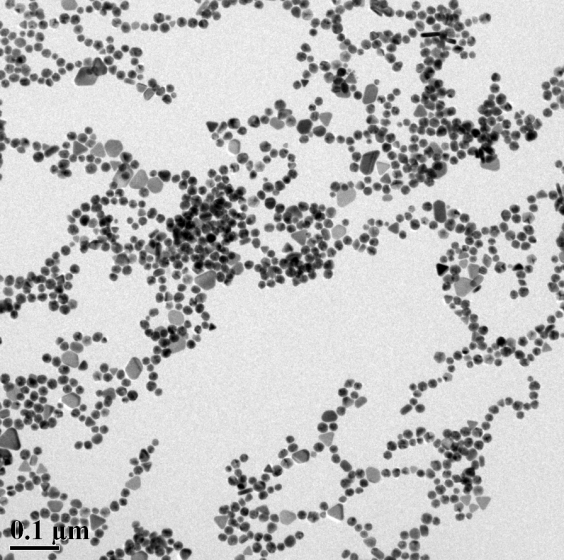

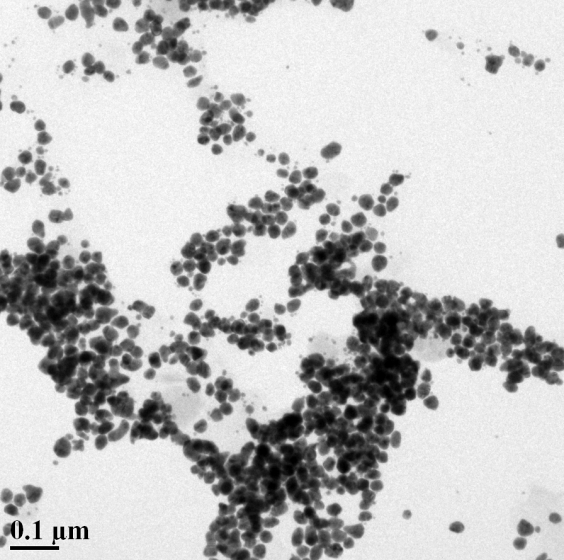
**

**(d)**

**(c)**

**Figure S1.** TEM images of gold nanoparticles on a larger scale in various cases. (a) citrate:AuCl_3_ of 2:1 @ pH-5, (b) citrate:AuCl_3_ of 5:1 @ pH-5, (c) citrate:AuCl_3_ of 2:1 @ pH-4 and, (d) citrate:AuCl_3_ of 2:1 @ pH-6. Particles are monodisperse in (a) and (b), anisotropic shapes are seen in (c) and agglomeration of particles is observed in (d).

**Figure S2.** (a) Surface plasmon resonance wavelength with initial pH of the solution of sodium citrate and gold chloride. (b) Existence of an optimal pH corresponding to each citrate to AuCl_3_ ratio.

**Figure S3.** Zeta potential of AuNPs synthesized under varying pH conditions (citrate:AuCl_3_ = 5:1).

Zeta potential of AuNPs synthesized at room temperature at various pH condition indicates the relative stability of AuNPs synthesized at pH=5 compared to other pH conditions (Figure S5).

**Figure S4.** Particle size distribution for citrate:AuCl_3_ ratio (a) 2:1 @ pH=5, (b) 2:1 @ pH=4, and (c) 5:1 @ pH=5. Particle size distribution could not be calculated for AuNPs synthesized under other precursor to reductant ratio and pH conditions due to extensive coalescence.
